# Linking microbial ecology to the cycling of neutral and acidic polysaccharides in pustular mats from Shark Bay, Western Australia

**DOI:** 10.3389/fmicb.2025.1684648

**Published:** 2025-10-13

**Authors:** Emilie J. Skoog, Elise Cutts, Tanja Bosak

**Affiliations:** ^1^Department of Earth, Atmospheric and Planetary Sciences, Massachusetts Institute of Technology, Cambridge, MA, United States; ^2^Scripps Institution of Oceanography, University of California, San Diego, La Jolla, CA, United States

**Keywords:** cyanobacteria, EPS, extracellular polymeric substances, microbial mat, carbohydrate cycling

## Abstract

Cyanobacteria and other microbes in peritidal microbial mats have produced extracellular polymeric substances (EPS) for more than two billion years. The production and degradation of EPS contributes to the biogeochemical cycling of carbon and carbonate precipitation within modern microbial mats, but key microbes involved in the cycling of EPS remain unidentified. Here, we investigate the cycling of EPS in the peritidal pustular mats of Shark Bay, Western Australia. We characterize the chemical composition of EPS produced by cyanobacterial enrichment cultures under natural and UV-stress conditions and link these findings to the metabolic potential for EPS production and degradation encoded in 84 metagenome-assembled genomes (MAGs) from the mat community. We further identify the key microbial degraders of specific acidic and neutral polysaccharides in this community by cultivating enrichment cultures on seven commercially available polysaccharides representative of those present in the mats and assessing the dominant taxa. All sequenced Cyanobacteria MAGs have the potential to synthesize mannose, fucose, glucose, arabinose, rhamnose, galactose, xylose, N-acetylglucosamine, galacturonic acid and glucuronic acid. Biochemical analyses confirm the presence of nearly all these monosaccharides in the hydrolysates of EPS extracted from UV- and non-UV exposed cyanobacterial enrichments. Ultraviolet radiation influences the structure and composition of EPS by reducing the hydration, potentially due to cross-linking among polymers in EPS and increasing the relative abundances of uronic acids and xylose in polysaccharides. Analyses of carbohydrate-active enzymes (CAZymes) in the MAGs and of 16S rRNA sequences from experimental polysaccharide enrichments point to major roles for Bacteroidetes, Planctomycetes, and Verrucomicrobia in the cycling of acidic EPS. These experiments reveal a complex interplay among microbial community composition, CAZyme diversity, environmental stressors, and EPS cycling, which together shape carbon flow and biomineralization in pustular mats in Shark Bay.

## 1 Introduction

Silicified pustular mats from peritidal marine environments contain the oldest known fossils of cyanobacteria encased in multiple layers of extracellular polymeric substances (EPS). These fossilized mats provide evidence that cyanobacteria have been producing complex EPS for over two billion years (Hoffman, 1976; Golubic and Hofmann, 1976; Moore et al., 2023). The hypersaline environment of Shark Bay, Western Australia, hosts some of the best contemporary analogs of these fossil pustular mats (Hoffman, 1976; Hoffman et al., 1974; Jahnert and Collins, 2013; Moore et al., 2020; Skoog et al., 2022). In Shark Bay, thick layers of EPS envelope aggregates of coccoidal cyanobacteria, contributing to the characteristic pustular morphology of these mats (Golubic and Hofmann, 1976). Changes in EPS composition throughout the mat are thought to contribute to the biogeochemical cycling of carbon, sulfur, oxygen, and calcium within these systems (Visscher et al., 1999; Richert et al., 2005; Gautret and Trichet, 2005; Decho et al., 2005; Braissant et al., 2009; Visscher et al., 2010; Stuart et al., 2016; Al Disi et al., 2019; del Buey et al., 2021; Skoog et al., 2022; Cutts et al., 2022).

EPS protects benthic microbial communities from environmental stresses including desiccation (Tamaru et al., 2005; Wright et al., 2005), UV radiation (Garcia-Pichel and Castenholz, 1991; Garcia-Pichel et al., 1992; Garcia-Pichel and Castenholz, 1993; Garcia-Pichel, 1998; Singh et al., 2023) and nutrient limitation (Pereira et al., 2009). Although the structure and composition of EPS are thought to play a crucial role in the formation and adaptation of microbial communities to shallow marine environments (Flemming, 2016; Decho and Gutierrez, 2017), EPS in the microbial mats from Shark Bay and most other peritidal environments have remained poorly characterized due to the structural complexity and difficulty in resolving the overall architecture of macromolecular mixtures. The EPS produced by certain marine and terrestrial cyanobacteria consist mainly of complex heteropolysaccharides that can contain hexose (i.e., glucose, galactose, mannose, and fructose), pentose (i.e., ribose, xylose, and arabinose), deoxyhexose (i.e., fucose and rhamnose), and acidic hexose sugars (i.e., glucuronic and galacturonic acid; Pereira et al., 2009). However, the compositional and structural diversities of EPS across cyanobacterial species and environments remain poorly constrained. Cyanobacterial EPS can include proteins, lipids, extracellular DNA, acetylated or otherwise modified amino sugar moieties and sugars modified by pyruvate, lactate, phosphate, sulfate, and carboxyl moieties (Sutherland, 1999; De Philippis and Vincenzini, 1998, 2003; De Philippis et al., 2001; Moore et al., 2021; Skoog et al., 2022; Huang et al., 1998; Nicolaus et al., 1999; Kumar et al., 2018; Cruz et al., 2020; Laroche, 2022). Other microbes in mats such as sulfate-reducing bacteria, Alphaproteobacteria, and Gammaproteobacteria can also play a role in the synthesis of EPS matrices (Kennedy and Sutherland, 1987; Ford et al., 1991; Braissant et al., 2007; Vu et al., 2009; Decho and Gutierrez, 2017), which complicates efforts to determine which cyanobacteria are responsible for specific mono- and polysaccharide components within biofilm communities. Microbes degrade EPS in such communities by utilizing carbohydrate-active enzymes (CAZymes) to hydrolyze specific poly- and disaccharides and can additionally employ specific enzymes to remove certain functional groups (Drula et al., 2022; Breton et al., 2006), enabling other microbial community members to use EPS-derived polysaccharides as a carbon source (Decho et al., 2005; Wong et al., 2018, 2020; Cutts et al., 2022). Previous studies have reported the presence and activity of CAZymes in microbial mats (Braissant et al., 2009; Ruvindy et al., 2016; Stuart et al., 2016; Wong et al., 2018; Skoog et al., 2022) and considered their impact on silicification and carbonate mineralization in these ecosystems (Arp et al., 2012; Suarez-Gonzalez and Reitner, 2021; Braissant et al., 2007, 2009; Pace et al., 2018; Moore et al., 2020, 2021, 2023; Skoog et al., 2022). Despite the long-recognized importance of EPS and the cycling of EPS in microbial mats, the specific microbial contributors to its synthesis, modification, and degradation remain poorly resolved. Addressing this gap is essential for linking EPS composition to microbial community structure, biogeochemical cycling, and the long-term resilience of these ecosystems.

Here, we explore the composition of EPS in a pustular microbial mat community from Shark Bay, Western Australia, and assess the ability of different community members to degrade this EPS and utilize structural analogs of polysaccharides present in the natural matrix. We hypothesize that cyanobacterial EPS composition shifts in response to UV stress, and that these changes both influence microbial community structure and selects for microbes possessing CAZymes capable of degrading cyanobacteria-derived polysaccharides. First, we characterize the metagenomic potential of cyanobacterial metagenome-assembled genomes (MAGs) from a pustular mat to synthesize and modify specific monosaccharides and polysaccharides. Next, we compare this potential to the composition of monosaccharides in hydrolyzed EPS from enriched cyanobacterial communities grown in the presence and absence of UV stress. Then, we examine the potential of 84 MAGs derived from a Shark Bay pustular mat to modify and degrade specific polysaccharides and relate these predictions to the composition of microbial communities enriched on seven different polysaccharides and EPS extracted from natural pustular mats.

## 2 Materials and methods

### 2.1 Metabolic capacities of cyanobacteria for EPS production and modification

**Figure 1:**
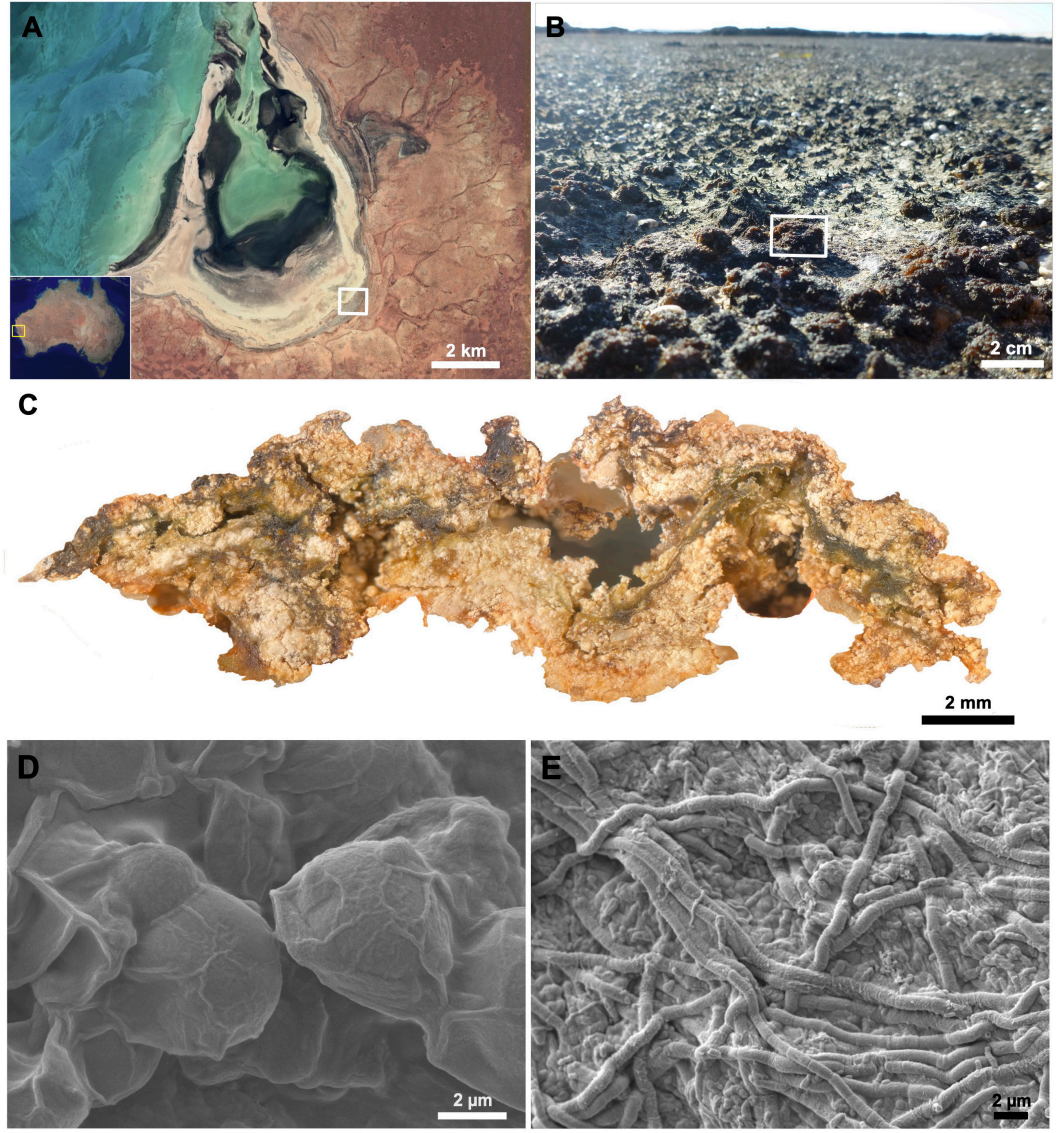
Macroscale to microscale view of a pustular mat community and associated cyanobacteria with their extracellular polymeric substances (EPS). Microbial mats were collected from **(A)** Shark Bay, Australia (yellow box) along the hypersaline, peritidal region of Carbla Beach (white box). **(B)** Pustular mats (white rectangle) were sampled, and a **(C)** cross-section of a pustular mat reveals a tight matrix of organo-sedimentary material formed and bound together by EPS produced by **(D)** coccoidal and **(E)** filamentous cyanobacteria. **(B)** Adapted from **(C)** from Skoog et al. (2022).

A pustular microbial mat sample from Shark Bay, Western Australia was collected and sequenced as previously described (Figures 1A, B; Skoog et al., 2022). Resulting DNA sequences were quality filtered, assembled, binned into 84 medium-to-high quality MAGs, and taxonomically classified as described in Skoog et al. (2022). Three of these MAGs—MAG 10, MAG 34, MAG 54—represented Cyanobacteria and were 98.6%, 95.2%, and 90.2% complete, respectively, according to CheckM analyses (Skoog et al., 2022). GTDB-tk classification identified MAG 10, MAG 34, and MAG 54 as Cyanobacteria belonging to the families *Phormidesmiaceae, Pseudophormidiaceae*, and *Rubidibacteraceae*, respectively (Skoog et al., 2022). Each of these MAGs was annotated using the Department of Energy (DOE) Joint Genome Institute Integrated Microbial Genomes (JGI IMG) Annotation Pipeline v.4.16.5 (Markowitz et al., 2007; Huntemann et al., 2015) to predict the types of polysaccharides produced by each of these Cyanobacteria.

### 2.2 Sampling and enrichments

Sampled pustular mats that were not sequenced were transferred into sterile plastic plant culture jars (BioExpress, 190 mL, 68 mm × 68 mm) that contained hypersaline BG11 medium (Allen et al., 2009; Goh et al., 2009; https://doi.org/10.17504/protocols.io.bkcmksu6). These pustular mat enrichments were exposed to 24 h of light at 21 °C. The hypersaline modified BG11 medium was replaced every 2 weeks to maintain the solution pH between 7.5 and 8.5. To enrich pustule-forming cyanobacteria from the mats, cyanobacterial pustules were repeatedly transferred between Petri dishes containing hypersaline BG11 medium solidified with agar (https://doi.org/10.17504/protocols.io.bkchkst6) and hypersaline BG11 liquid medium. These enriched pustules were used in experimental comparisons to characterize EPS produced by cyanobacteria either exposed or not exposed to UV light. For the non-UV treatment (“Cyano”) samples, these enrichments were used to inoculate three cyanobacterial enrichment cultures grown in hypersaline BG11 liquid medium under natural (LED) light at 21 °C for 24 h over 3 weeks. To assess changes in EPS composition under UV stress, pustules were also used to inoculate three enrichment cultures (termed “UV-Cyano”) grown in hypersaline BG11 medium under both UV-A irradiation (315–400 nm; ~35–50 W/m^2^ at the culture surface, continuous exposure) and natural (LED) light at 21 °C for the same duration of time.

### 2.3 EPS visualization

To visualize the EPS produced by cyanobacteria, small portions of the cyanobacterial enrichment cultures (i.e., “Cyano” cultures) were filtered onto 0.2 μm polycarbonate membrane filters and allowed to air dry overnight. Dried samples were affixed onto an SEM mounting stage with double-coated carbon conductive tape (Ted Pella Inc., Product #16084-7, Redding, CA, USA), sputter coated with 5 nm carbon coating, and visualized with a JEOL 7900F scanning electron microscope (SEM) at the Harvard Center for Nanoscale Systems (CNS).

### 2.4 EPS extraction

To characterize the sugars in Cyano and UV-Cyano cultures, EPS was extracted from dime-sized portions of each sample. The samples were pelleted by centrifugation for 1 min at 3580 × g (rotor radius 20 cm), immersed in 2.5 mL of 0.25 M NaCl solution, incubated at 60 °C overnight, and centrifuged for 1 min at 3580 × g (rotor radius 20 cm). The supernatants were then transferred to ice-cold 200 proof ethanol in a 1:1 ratio in sterile test tubes and the mixtures were incubated at 4 °C overnight. Remaining pellets were re-immersed in 2.5 mL of 0.25 M NaCl solution and the process was repeated to extract any additional EPS from each culture. The gelatinous extract from the solution was pelleted by centrifuging for 5 min at 14,310 × g (rotor radius 20 cm) at 21 °C. The resulting pellets were air-dried overnight in a biosafety hood.

### 2.5 EPS protein concentration analyses

The Modified Lowry Protein Assay Kit (ThermoScientific, cat#23240) was used to determine total protein concentration in the extracted EPS. Briefly, albumin (BSA) standards were dissolved in milliQ water at concentrations of 0, 1, 5, 25, 125, 250, 500, and 1,000 μg/mL. Samples of extracted EPS (20 mg dry weight) from cyanobacterial enrichment cultures were diluted in milliQ water to produce dilutions of 0.125, 0.25, 0.5, 1, and 2 g/L of EPS. 1.0 milliliter of Modified Lowry Reagent was added to 200 μL aliquots of these solutions in 1.5 mL Eppendorf^®^ microtubes (Eppendorf North America, NY, USA, cat#022364111) and incubated at room temperature for 10 min. Subsequently, 100 μL of prepared 1X Folin-Ciocalteu Reagent was added to each tube and immediately vortexed before incubating in the dark for 30 min. 200 microliters of each solution was added to separate wells of a 96-well plate in triplicate and their absorbances were measured at 750 nm using a BioTek microplate reader (BioTek, Synergy 2, Winooski, VT, USA). The spectra were analyzed by BioTek Gen5 Data Analysis software.

### 2.6 EPS characterization

EPS was characterized using high-performance anion exchange chromatography/pulsed amperometric detection (HPAEC-PAD) at CarbExplore Research (Groningen, The Netherlands). All EPS samples and standards were dissolved in ultrapure water or 2M TFA to a concentration of 1 mg/mL. Galactose, glucose, dextrose, fructose, fucose, mannose, fucoidan (sulfated), xylan, chitin, chondroitin sulfate, maltose, arabinose, xylose, rhamnose, glucuronic acid, galacturonic acid, N-acetyl glucosamine, and high-molecular weight dextran were used as standards. Water solubilized samples and standards were analyzed by HPAEC-PAD, with a quantification limit of 10 μM, for the assessment of the free monosaccharides present in the samples. Samples and standard solubilized in TFA were also hydrolyzed and subsequently analyzed using HPAEC-PAD. Standards were diluted 10 times before injections. Additionally, Fourier transform infrared (FT-IR) spectra (4,000–600 cm^−1^) of UV-exposed and non-UV-exposed cyanobacterial EPS pellets were acquired on a Thermal Scientific Nicolet™ iS50 FT-IR Spectrometer at the MIT Materials Research Laboratory.

### 2.7 Carbohydrate-active enzyme annotation and substrate assignment

To assess the genomic potential for the degradation of polysaccharides in the microbial community, we annotated CAZymes in all 84 medium-to-high quality MAGs derived from the Shark Bay pustular mat (Skoog et al., 2022). Glycoside hydrolase (GH) and polysaccharide lyase (PL) genes were annotated according to the classification scheme of the CAZy database (Drula et al., 2022) using a local installation of the dbCAN2 web server annotation pipeline (Zhang et al., 2018). The pipeline integrates three tools to identify CAZymes: HMMER3 (Eddy, 2011) to search against the dbCAN CAZyme HMM database (Yin et al., 2012), DIAMOND (Buchfink et al., 2021) to find Blast hits to the CAZy database, and a Hotpep (Busk et al., 2017) search for sort conserved CAZyme motifs. All searches were performed with default parameters using the run_dbCAN 2.0.6 Python package (https://github.com/linnabrown/run_dbcan). CAZymes were assigned to families based on the agreement of at least 2 of the 3 search tools (Zhang et al., 2018).

GH and PL families were linked to predicted functions and substrates by manually assessing the categories of substrates (e.g., alpha-glucan) and bonds (e.g., beta-(1,3) glucose) targeted by each family. To start, specific substrates [e.g., chitin, beta-(1,4)-glucan] and activities [e.g., endohydrolysis of alpha-(1,4) bonds between glucose monomers] were assigned to EC numbers as provided by the CAZyme database. These manual associations were then used to populate CAZyme families with substrates and activities based on their EC numbers (Supplementary Tables 1, 2). Substrate and activity assignments were then refined by cross referencing the automatically-generated assignments against the CAZydb activity description strings. Sets of categories for substrates (i.e., alpha-glucanase) and bond activity (alpha glucosidase) were defined to more succinctly summarize the potential activities observed in the dataset (Supplementary Tables 2, 3). GH and PL were considered polyspecific if they had predicted activity on more than 4 different specific glycosidic bonds or 9 different substrates.

### 2.8 Enrichment experiments

To identify key taxa involved in degrading different types of polysaccharides and native EPS, dime-sized fragments of Shark Bay pustular mats were broken apart to form a “pustule slurry” and incubated with a variety of polysaccharide substrates for 2 weeks in the dark. The resulting shifts in community composition were quantified by 16S rRNA gene amplicon sequencing. Specifically, samples of pustular mats were incubated for 2 weeks with a 12:12 h light:dark cycle. Following the initial incubation period, the light-grown pustules were transferred into a 15 mL Falcon tube, suspended in 5 mL of the fresh hypersaline BG11 medium and homogenized into a slurry using a sterile metal spatula. Triplicate sterile 50 mL glass serum bottles containing 20 mL of BG11 medium amended with one of several polysaccharides—cellulose (1% w/v), agar (0.5% w/v), chondroitin sulfate (0.5 w/v), laminarin (1% w/v), xylan (0.5% w/v), chitin (1% w/v), and pectin (0.5% w/v)—were inoculated by 200 μL of pustule slurry. These cultures were capped with sterile foam plugs and incubated in a shaker incubator in the dark for 2 weeks before sampling. More than half of the cultures were visibly turbid at this point. The cultures that were not amended by specific polysaccharides were incubated with a 12:12 h light:dark cycle to continue to enrich for microbes that grow on cyanobacterially-produced EPS (“slurry”). Each condition was set up in triplicate, and after 2 weeks, 1.5 mL of medium was sampled from each serum bottle and the samples were stored at −80 °C for 3 months.

Whole genomic DNA was extracted from the samples and from ~0.5 cm diameter chips of the thawed pustular mat material (“pustule”) using Powersoil^®^ DNA Isolation Kit (MO BIO Laboratories, Inc Carlsbad, CA, USA). DNA yield was evaluated using a Qubit 2.0 Fluorometer (Thermo Fisher Scientific, Chino, CA, USA). The extracted whole genomic DNA was submitted to the BioMicro Center Core Facility at MIT for 16S-V4 SSU amplicon (primers: U515F GTGCCAGCMGCCGCGGTAA, E786R GGACTACHVGGGTWTCTAAT) sequencing on an Illumina MiSiq sequencer with a V3 (2x150 BP) kit. Demultiplexed reads were provided by the BioMicro Core Center and further processing of the sequencing data was performed using R (v4.1.2). The first 20 bp of each read were trimmed to remove the non-biological fraction of the reads. Amplicon sequence variants (ASVs) were inferred using the dada2 package (V1.22.0; Callahan et al., 2016). Taxonomic annotation of the ASVs was done with the RDP implementation in the dada2 package using the silva non-redundant v138.1 train set. Ampvis2 (v2.7.17; Andersen et al., 2018) was subsequently used to analyze and visualize the data.

Principal Coordinates Analysis (PCoA) plots were used to visualize the differences in microbial community composition across pustules, slurry, and enrichment cultures. Bray-Curtis distances were calculated from all ASVs using the vegdist() function in the vegan R package. Ordination was conducted using classical multidimensional scaling [cmdscale()], and the first two principal coordinates were used for visualization. The percent variance explained by each axis was determined from the eigenvalues of the ordination. The resulting PCoA plot was generated in ggplot2, with colors representing experimental conditions to highlight community differences. Differences in community composition among enrichment conditions were tested using PERMANOVA (adonis2, vegan R package) with 999 permutations on Bray-Curtis dissimilarities. Pairwise comparisons were adjusted using the Benjamini-Hochberg false discovery rate to obtain q-values for differences between specific enrichment groups.

## 3 Results

### 3.1 Metagenomic potential of EPS production and modification by cyanobacteria

**Figure 2:**
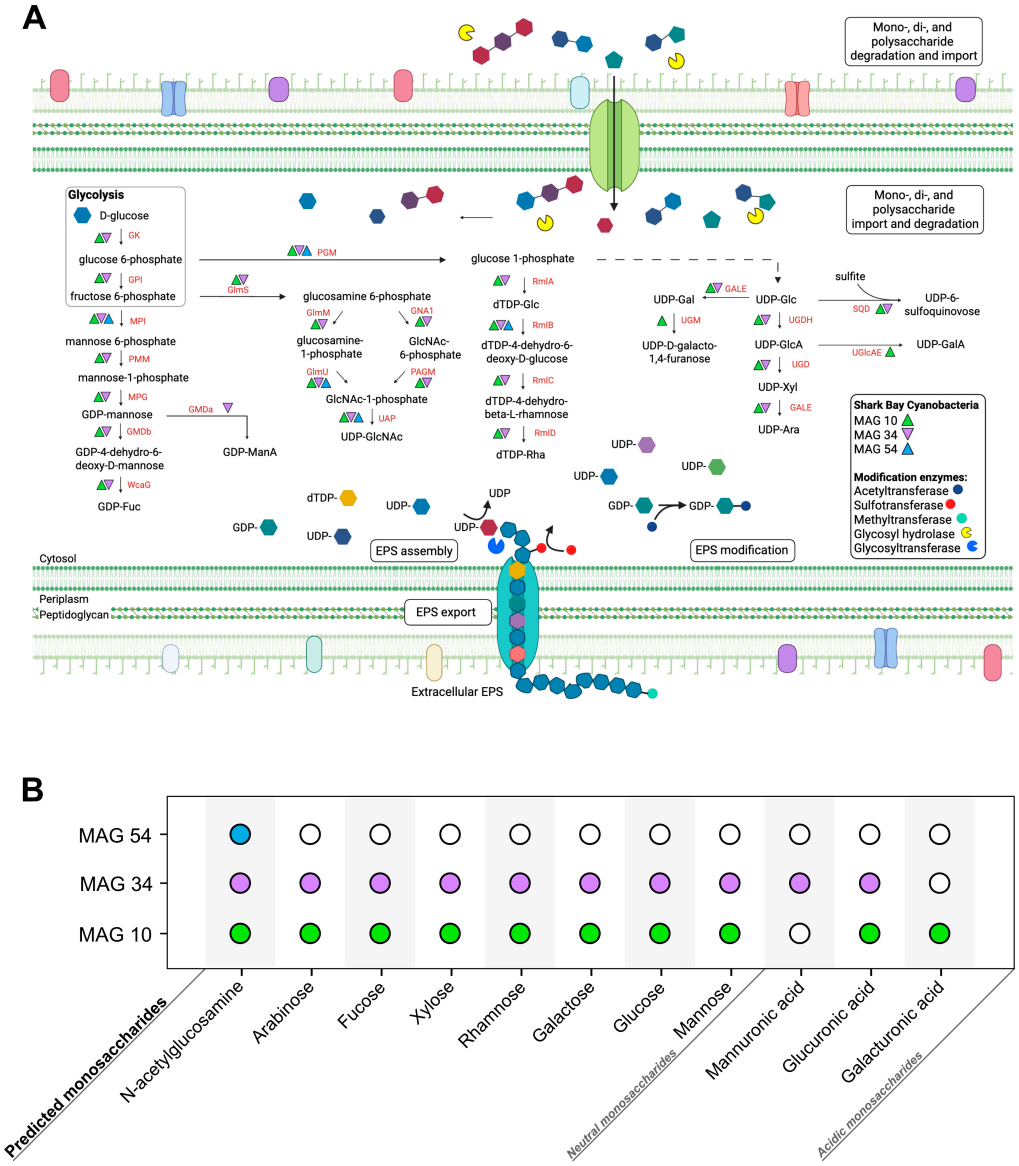
Predicted monosaccharide production by Cyanobacteria in pustular mats from Shark Bay. **(A)** Genes involved in the production and modification of various monosaccharides within the three Cyanobacteria MAGs from Shark Bay. Monosaccharides are first activated by NDPs (e.g., UDP, GDP, dTDP) to form NDP-sugars; these activated monosaccharide precursors are then assembled into di- and polysaccharides by glycosyltransferases and exported out of the cell (Freeze and Elbein, 2009). Monosaccharide abbreviations: GlcA, Glucuronic acid; GalA, galacturonic acid; ManA, mannuronic acid; GlcNAc, N-acetylglucosamine; Fuc, Fucose; Glu, Glucose; Gal, Galactose; Xyl, Xylose; Ara, Arabinose; Rha, Rhamnose. Enzyme abbreviations: GK, Glucokinase; MPI, Mannose-6 phosphate isomerase; GPI, Glucose-6-phosphate isomerase; PGM, Phosphoglucomutase; PMM, Phosphomannomutase; MPG, Mannose-1-phosphate guanylyltransferase; GMDa, GDP-mannose 6- dehydrogenase; GMDb, GDP-mannose 4,6-dehydratase; WcaG, GDP-L-fucose synthase; RmlA, Glucose- 1-phosphate thymidylyltransferase; RmlB, dTDP-glucose 4,6-dehydratase; RmlC, dTDP-4- dehydrorhamnose 3,5-epimerase; RmlD, dTDP-4-dehydrorhamnose reductase; UMG, UDP-galactopyranose mutase; GALE, UDP-glucose 4-epimerase; SQD, UDP-sulfoquinovose synthase; UGDH, UDP-glucose 6- dehydrogenase; UGD, UDP-glucuronate decarboxylase; UGlcAE, UDP-glucuronate 4-epimerase; GlmS, Glucosamine:fructose-6-phosphate aminotransferase; GNA1, N-acetylglucosamine-6-phosphate deacetylase; GlmM, phosphoglucosamine mutase; GlmU, Glucosamine-1-phosphate N-acetyltransferase; PAGM, Phosphoacetylglucosamine mutase; UAP, UDP-GlcNAc pyrophosphorylase. **(B)** Monosaccharides predicted to be synthesized by each cyanobacterial MAG. Solid circles represent the predicted production potential of each MAG, with each MAG colored uniquely.

Coccoidal and filamentous cyanobacteria that build pustular mats produce EPS and mucilaginous sheaths (Figure 1). These microbes were represented by three MAGs: MAG 10 (*Phormidesmiaceae*), MAG 34 (*Pseudophormidiaceae*), and MAG 54 (*Rubidibacteraceae*; Skoog et al., 2022). These MAGs encode the potential to produce a diverse range of monosaccharides—including glucose, galactose, fucose, mannose, xylose, arabinose, rhamnose, N-acetylglucosamine, galacturonic acid, glucuronic acid, and mannuronic acid (Figure 2; Supplementary Figure 1) and the genomic capacity for synthesizing trehalose, a compatible solute (Supplementary Figure 1). The three MAGs encode multiple genes involved in the production and modification of EPS (Figure 2; Supplementary Figure 1), but *Rubidibacteraceae* (MAG 54) surprisingly lacks most genes involved in polysaccharide biosynthesis (Figure 2) even though MAG 54 represents coccoidal cyanobacteria that create pustules and contain multilayered envelopes (Figure 1).

Glycosyltransferases (GTs) catalyze the formation of glycosidic bonds, linking together monosaccharide subunits to synthesize polysaccharide chains. All three cyanobacterial MAGs encoded at least one GT, spanning a diverse array of catalytic activities, including sucrose synthase, cellulose synthase, chitin synthase, mannosyltransferase, glucosyltransferase, galactosyltransferase, and rhamnosyltransferase (Supplementary Figure 1). Additionally, genes encoding polysaccharide-modifying enzymes such as acetyltransferases, methyltransferases, and sulfotransferases suggest further compositional diversity in extracellular polysaccharides, particularly in MAG 10 and MAG 34 (Supplementary Figure 1). MAG 34 harbors the most extensive repertoire of genes for polysaccharide biosynthesis and modification, whereas MAG 54 lacks most of these genes. Consequently, the composition of EPS in mats, including monosaccharide profiles and surface group modifications, likely depends on the relative abundances and activities of different cyanobacteria. While cyanobacterial MAGs contain the highest number and diversity of GTs and are the key players in EPS synthesis, other MAGs within the community also harbor GT and polysaccharide export genes (Skoog et al., 2022), suggesting that multiple organisms may contribute to modifying and shaping EPS composition. This combined genetic potential across Cyanobacteria and other MAGs predicts a wide range of polysaccharides in the EPS of pustular mats.

### 3.2 Chemical characterization of EPS

Previous studies have identified sulfated polysaccharides in pustular mats from Shark Bay, characterized EPS produced by cyanobacteria enriched from these mats (Moore et al., 2021; Skoog et al., 2022) and reported an increase in EPS production and formation of multilayered envelopes around the coccoidal, pustule-forming cyanobacteria grown in the presence of UV radiation (Moore et al., 2023). These cyanobacterial responses to stress and the large predicted potential diversity of polysaccharides in cyanobacterial EPS suggests that the composition of the EPS around pustules may change as a function of stress and community composition and activity. We therefore characterized the EPS of cyanobacterial enrichment cultures grown in the presence and absence of UV radiation to look for such changes.

**Figure 3:**
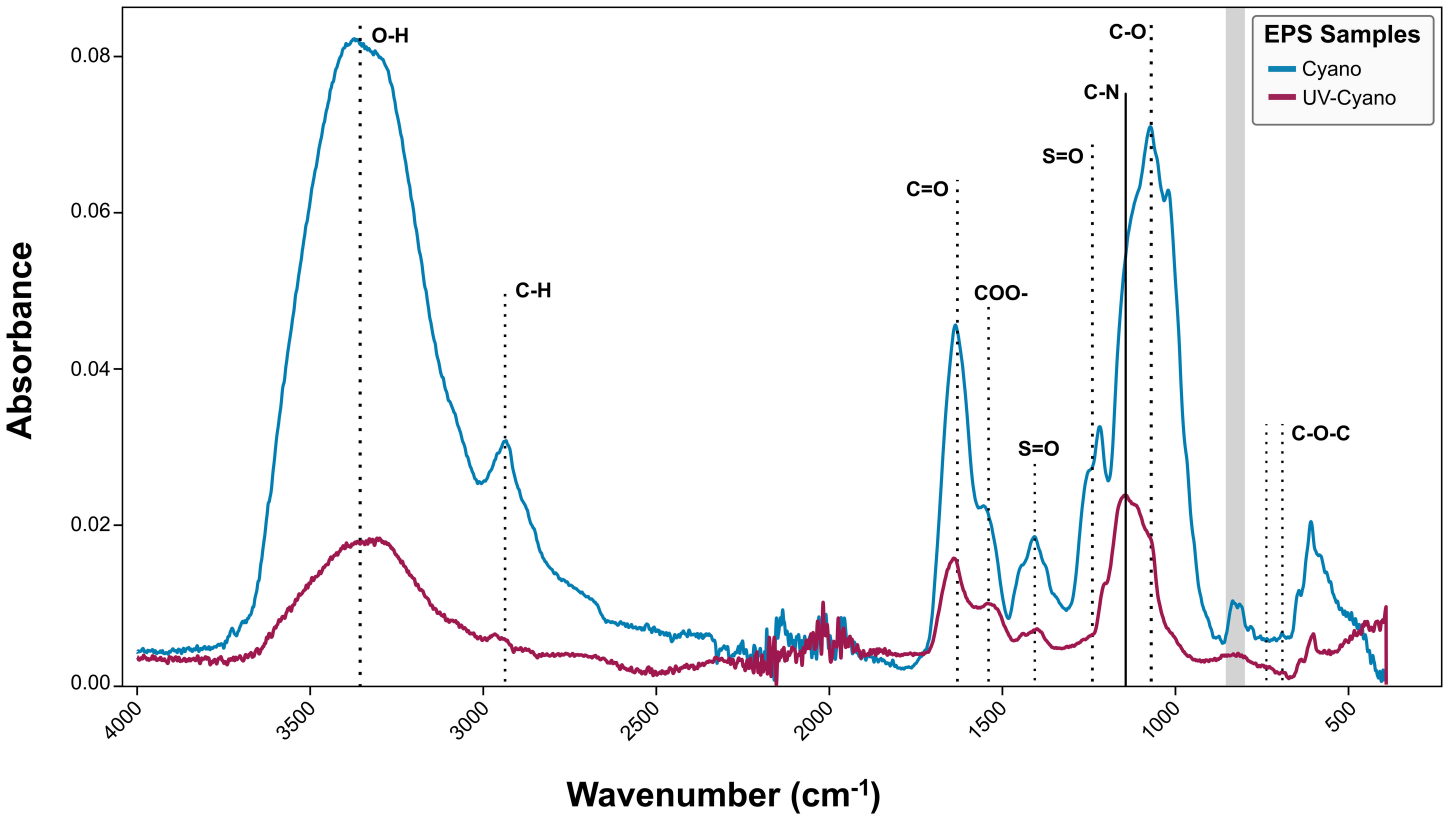
FT-IR analysis of Shark Bay EPS extracted from cyanobacterial enrichment cultures grown under natural light (blue) and cyanobacterial enrichment cultures grown under UV light (maroon).

Microscopic imaging of the cultures grown in the presence and absence of UV radiation confirmed that coccoidal cyanobacteria remained the primary producers under both conditions. Measurements of the protein content and FT-IR characterization of all extracted EPS provided chemical evidence for the changes in protein content and the structure and composition of polysaccharides as a function of the community composition and UV stress (Figure 3). Dry EPS extracted from the Cyano and UV-Cyano samples contained approximately 0.15%, and 1% of the total dry weight as protein, respectively. Absolute EPS yields were measured at 0.51 mg EPS per 2 g biomass for Cyano cultures and 0.39 mg EPS per 2 g biomass for UV-Cyano cultures. Because dried EPS masses were so low, biological triplicates were pooled per condition for compositional analyses.

The EPS from Cyano and UV-Cyano exhibited common spectral features, including a strong, broad O–H and secondary carboxylic acid amide stretching (3,200–3,550 cm^−1^), C–H stretching (2,840–3,000 cm^−1^), broad amide I and carbonyl bands from 1,600 to 1,650 cm^−1^ with the secondary amide around 1,530–1,550 cm^−1^, the carboxylate band at 1,410 cm^−1^, the C–O stretching vibrations at 1,078 cm^−1^, the broad glycosidic C-O-C linkage in carbohydrates at 1,010–1,080 cm^−1^, and the weaker modes in the 500–900 cm^−1^ region that are indicative of polysaccharides, but less amenable to definitive assignments (Figure 3; Gilli et al., 1994). The Cyano sample exhibited strong common spectral features, with an additional weak 1,730 cm^−1^ shoulder of the stretching vibration of C-O aldehyde (Smith, 2011) and multiple sulfate ester (S-O) peaks in the 1,210–1,260 cm^−1^ region, a peak at 1,020 cm^−1^ tentatively assigned to S–O, and absorptions at 805–840 cm^−1^ tentatively assigned to C–O–S in a manner similar to various sulfated polysaccharides (Figure 3; Chopin et al., 1999; Gómez-Ordóñez and Rupérez, 2011). EPS from the UV-Cyano culture exhibited much weaker signals of hydroxyl groups and carboxyl bands than Cyano EPS. The weaker spectral signals of sulfate groups in the UV-Cyano sample, both relative to the amide I peak and to the -OH band, suggested a lower content of sulfated polysaccharides in cyanobacterial cultures exposed to UV stress.

**Figure 4:**
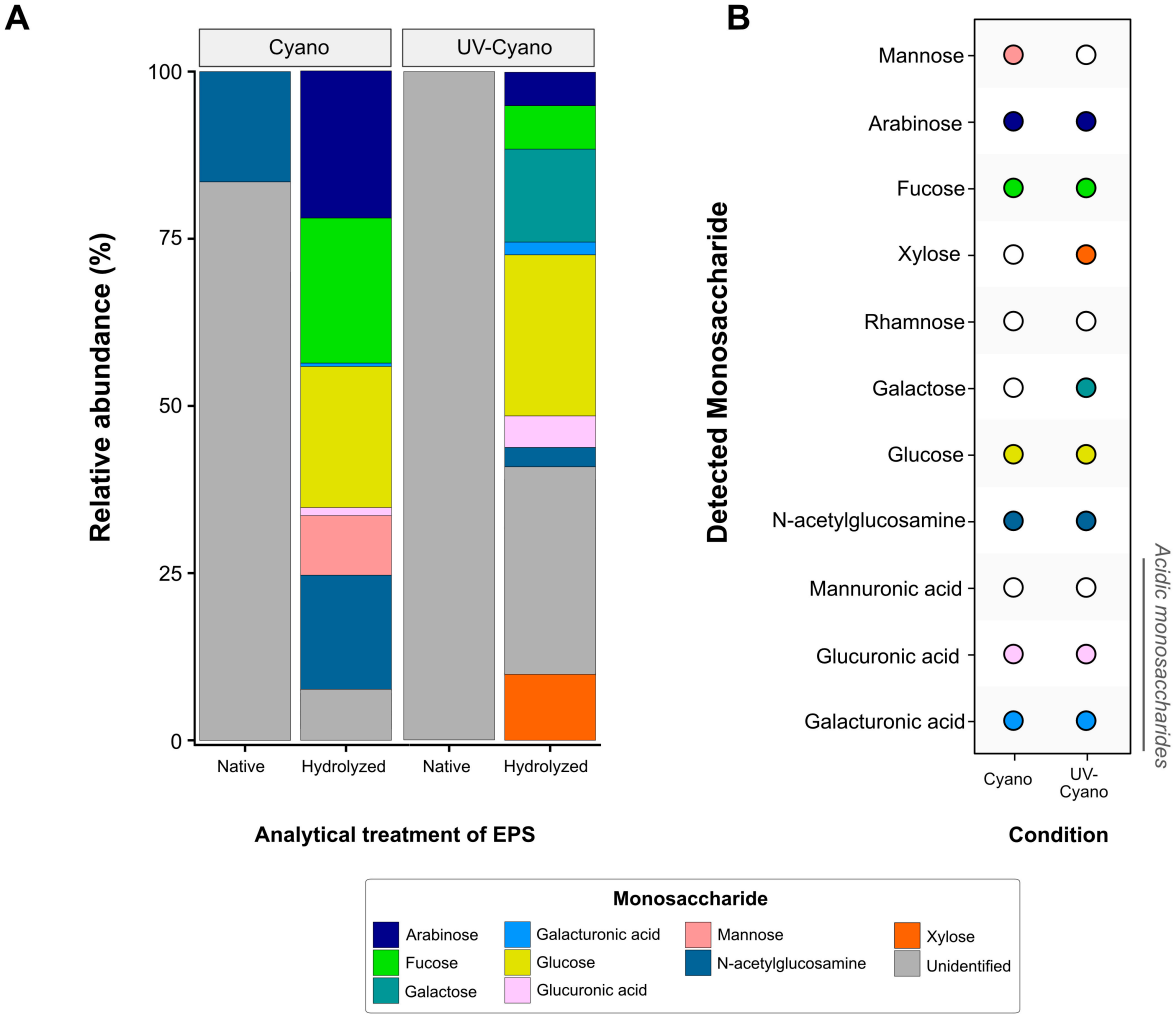
Observed monosaccharide production in Shark Bay enrichment cultures. **(A)** Relative abundance of specified monosaccharides in native and hydrolyzed EPS samples extracted from cyanobacterial enrichment cultures grown under natural light (Cyano) and cyanobacterial enrichment cultures grown under UV light (UV-Cyano). **(B)** Summary of observed monosaccharide production (solid circle colored according to monosaccharide) in each enrichment culture from Shark Bay.

We hypothesized that some of these differences might reflect changes in the composition of polysaccharides and tested this by analyzing monosaccharides in EPS by HPAEC-PAD. The analyses of native, unhydrolyzed EPS identified free N-acetylglucosamine in the Cyano sample (Figure 4), consistent with the detection of amide and acetyl absorptions by FTIR. Native EPS from UV-Cyano contained no identifiable monosaccharides (Figure 4). Hydrolysis of EPS enabled the identification of additional monosaccharides in each sample: both samples contained fucose, glucose, N-acetylglucosamine, galacturonic acid and glucuronic acid, supporting genomic predictions (Figures 2, 4). Galactose and xylose were only present in the EPS extracted from the UV-Cyano sample while mannose was only identified in EPS extracted from the Cyano sample (Figure 4). Several unidentified components (i.e., peaks 1, 2, 4, 12, 13, 14, 15, 16, and 17) were also present at low concentrations (< 10 μM) in both samples (Supplementary Table 4). Hydrolyzed EPS from the UV-Cyano culture contained the largest percentage of such unidentified compounds (31.2%; Supplementary Table 5). While the composition of these peaks cannot be determined with certainty, their relative abundance likely contributes to the infrared spectral signatures of the bulk material and to the observed differences between UV-Cyano and Cyano EPS (Figure 3). These unidentified peaks could potentially arise from monosaccharides modified by sulfate, acetyl, methyl, carboxyl or other functional groups. For example, some overlap between EPS hydrolysates and fucoidan and chondroitin sulfate standards suggests that these peaks could include modified monosaccharides, although their precise identities remain unresolved (Supplementary Figure 2). These findings show that EPS produced by pustule-forming cyanobacteria contains at least ten monosaccharide monomers that are modified by at least two charged moieties and change in response to UV exposure.

### 3.3 Community potential for the degradation of polysaccharides

**Figure 5:**
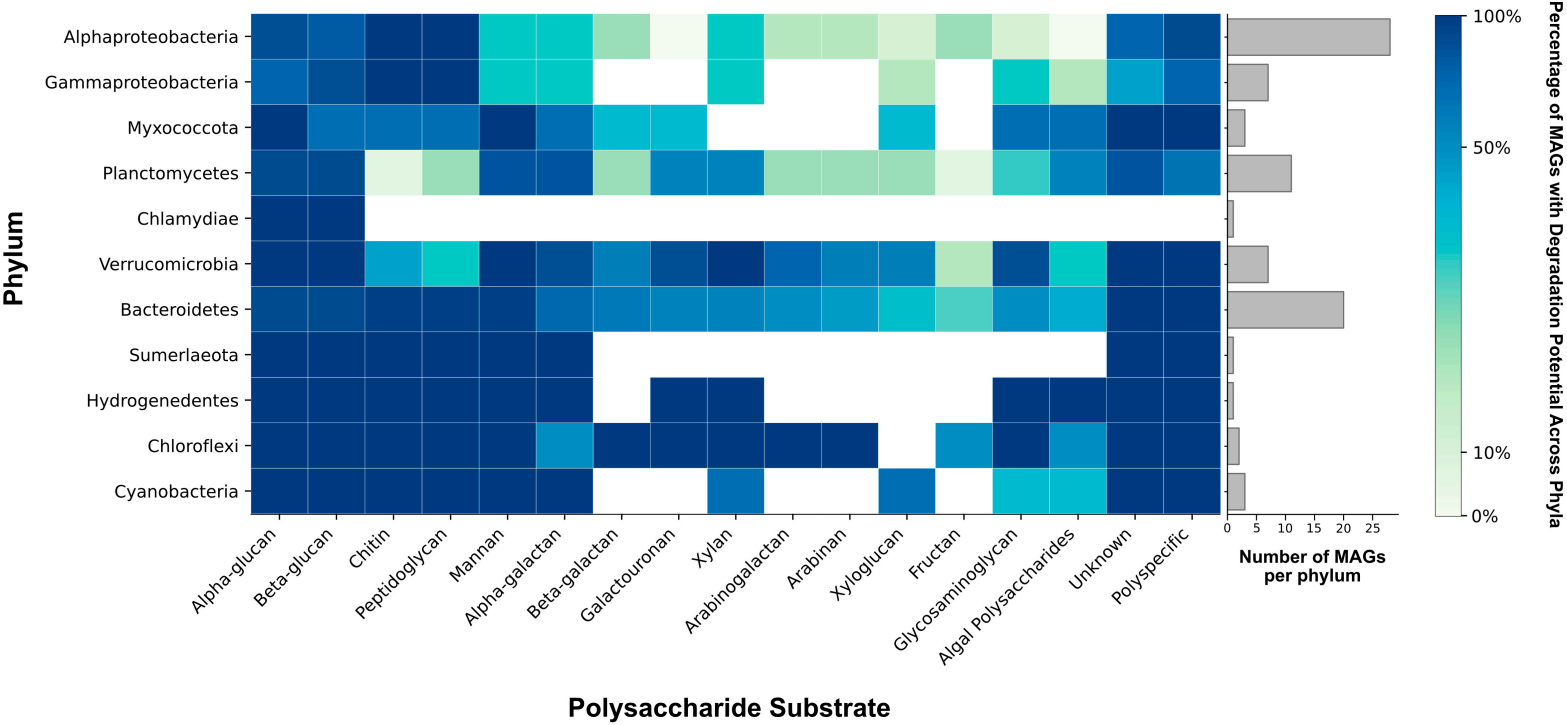
Phylum-wide distribution of polysaccharide degradation potential. Colors reflect the percentage of MAGs within each phylum that possess GH and PL genes necessary for the degradation of each polysaccharide substrate. The bar chart illustrates the number of MAGs represented in each phylum. Genes from polyspecific families were counted only in the column labeled “polyspecific” (see Methods). GH and PL with unknown activities are shown in the column labeled “unknown.” Algal polysaccharides include algin, carrageenan, ulvan, fucoidan, and beta-(1,4)-galacturonan. Glycosaminoglycans (GAGs) include chondroitin sulfate, dermatan sulfate, heparin/heparan sulfate, and unidentified GAGs.

Pustular mats host diverse microbial communities capable of degrading and metabolizing the EPS primarily produced by these cyanobacteria. Previously, we assessed the potential of MAGs from natural pustular mats to degrade sulfated polysaccharides and demonstrated the activity of sulfatases in the EPS from undegraded cyanobacterial pustules (Skoog et al., 2022). Because sulfated polysaccharides are only one component of EPS in pustular mats (Figure 3; Skoog et al., 2022), here we sought to examine the potential of the pustular mat community to degrade specific polysaccharides by mapping the identified major CAZymes in each MAG from this community to their predicted substrates (Figure 5; Supplementary Figure 3; Supplementary Table 6).

The major CAZyme families responsible for the degradation of polysaccharides are glycoside hydrolases (GHs) and polysaccharide lyases (PLs). GHs hydrolyze glycosidic bonds between sugars and PLs specifically cleave the glycosidic bonds of uronic acid-containing polysaccharides (Jedrzejas, 2000; Cantarel et al., 2009). Nearly all 84 MAGs from the pustular mat community had the potential to degrade either alpha- or beta-glucans (Figure 5; Supplementary Figure 3). Alphaproteobacteria, Gammaproteobacteria and some Bacteroides and Myxococcota were enriched in CAZymes that enabled the degradation of both chitin and peptidoglycan (Figure 5; Supplementary Figure 3). Chloroflexi, some Verrucomicrobia and Bacteroidetes, and one Alphaproteobacteria MAG (MAG 22) were enriched in CAZymes involved in the metabolism of xylan, arabinan, and arabinoglucan (Figure 5; Supplementary Figure 3). Trehalase (GH13) enzymes involved in the degradation of trehalose were identified in Cyanobacteria, Verrucomicrobia, Bacteroidetes, Alphaproteobacteria and Planctomycetes. MAGs from all phyla contained few CAZymes involved in the degradation of xyloglucan and fructan (Figure 5; Supplementary Figure 3), in keeping with the absence of fructose from all EPS hydrolysates and the detection of xylose only in the hydrolysate of UV-grown cyanobacterial enrichments (Figure 4). Most CAZymes responsible for the degradation of complex polysaccharides enriched with sulfate moieties and uronic acids—such as glycosaminoglycans (e.g., chondroitin sulfate, dermatan sulfate, and heparin/heparan sulfate) and algal polysaccharides (e.g., carrageenan, ulvan, and fucoidan)—were enriched in phyla belonging to Bacteroidetes, Hydrogenedentes, Chloroflexi, Planctomycetes, and Verrucomicrobia (Figure 5; Skoog et al., 2022). MAGs from the same phyla also encoded for most enzymes that can degrade polysaccharides containing uronic acids including galacturonans (Figure 5). Thus, microbes from these phyla in pustular mats are capable of degrading complex acetylated and acidic polysaccharides. In contrast, only ~14% of Alphaproteobacteria and Gammaproteobacteria MAGs contained a GH or PL predicted to act on sulfated glycosaminoglycans, galacturonan, or algal polysaccharides (Figure 5; Supplementary Figure 3). About 10% of GH and PL were unidentified, suggesting an even greater variety of possible carbohydrate degradation functions in Shark Bay mats than reported here.

### 3.4 Microbial communities enriched on different polysaccharides

**Figure 6:**
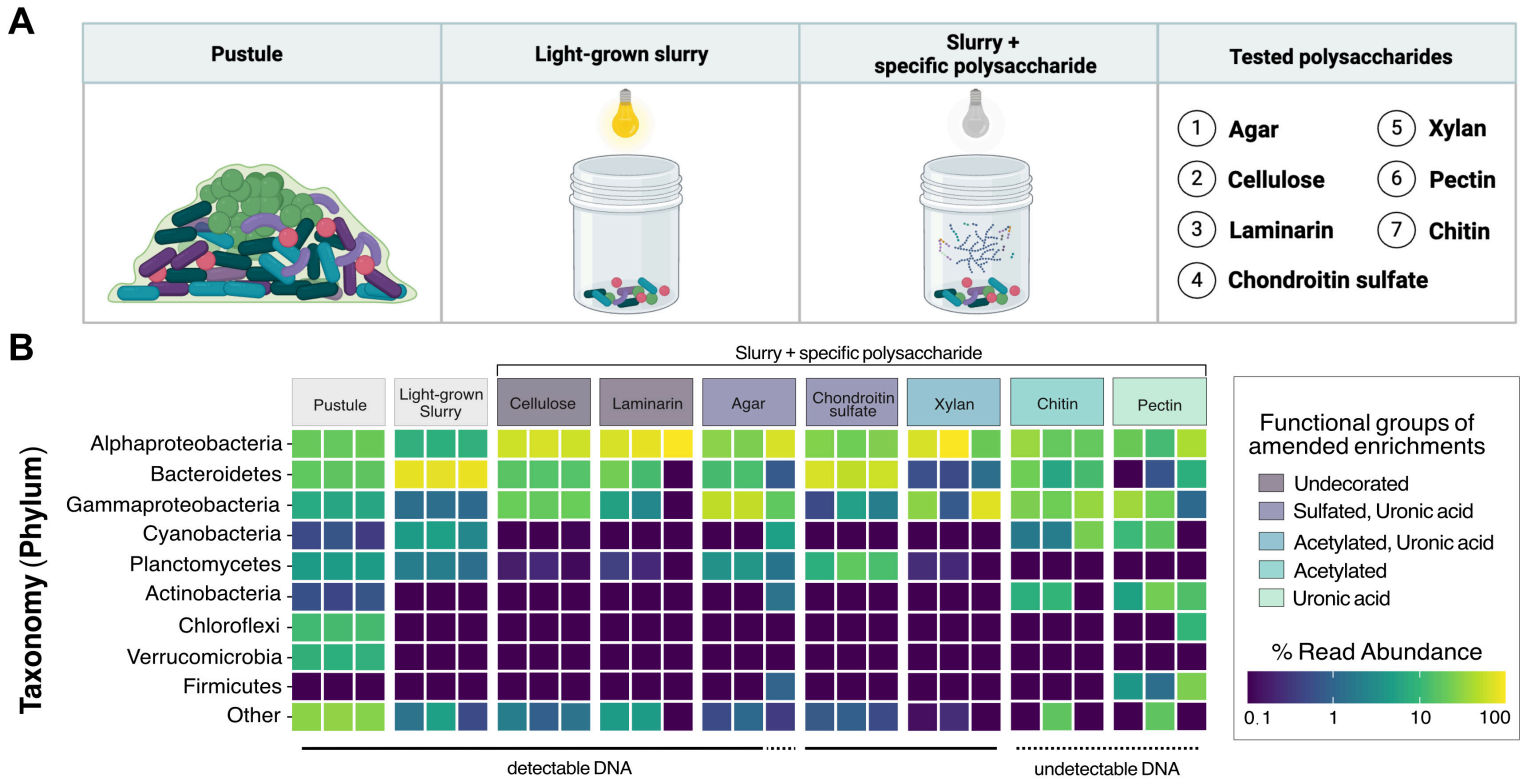
16S rRNA analysis of polysaccharide-enriched cultures derived from a Shark Bay pustular mat. **(A)** Schematic representation of cultured samples used to assess microbial community degradation of specific polysaccharides. **(B)** Relative abundances of ASVs in cultures supplemented with polysaccharides categorized by their functional groups. Each tile represents one biological replicate.

**Figure 7:**
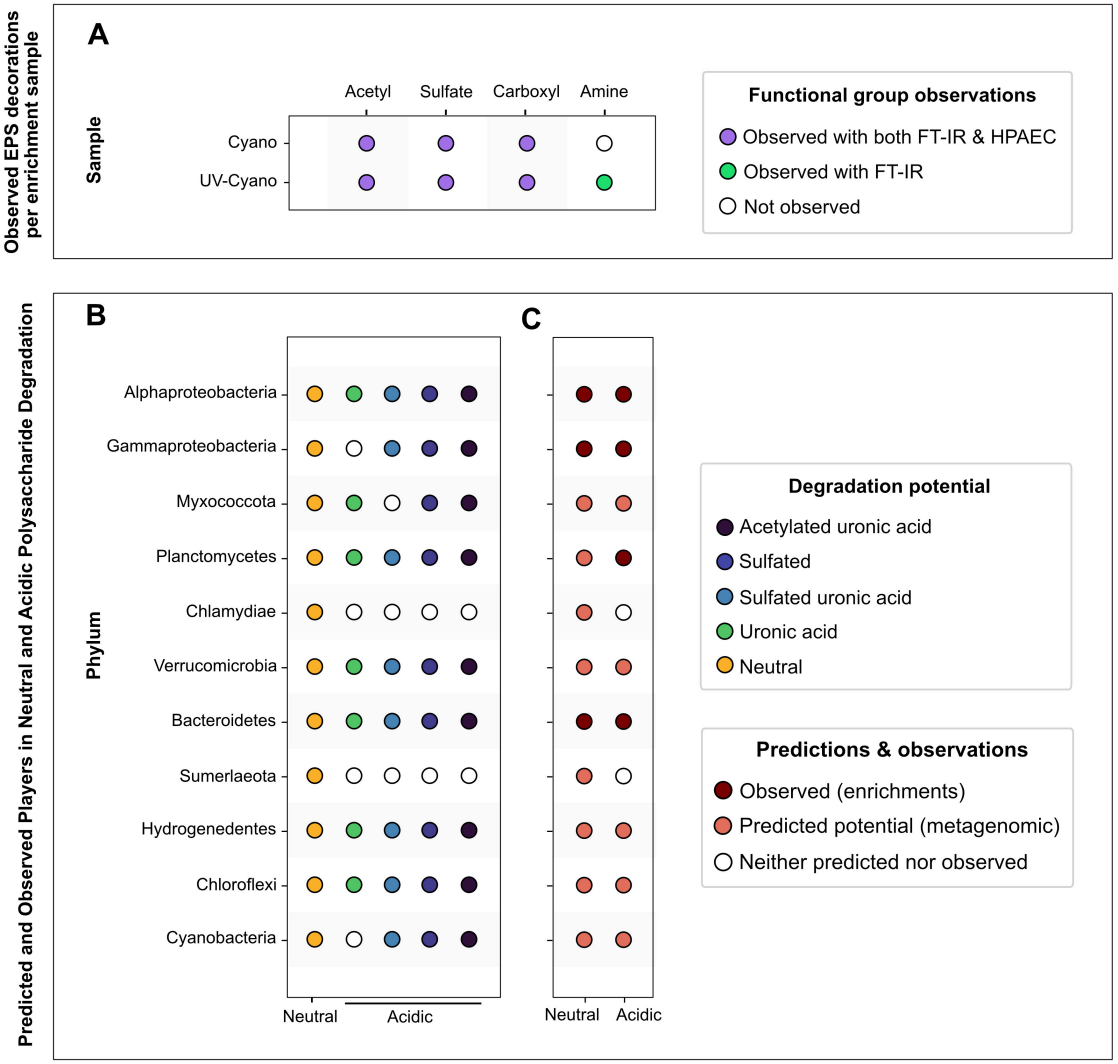
Overview of polysaccharide modifications and microbial degraders of neutral and acidic polysaccharides in a pustular mat. **(A)** Summary of observed functional groups identified with HPAEC-PAD and FT-IR detection methods per enrichment sample. **(B)** Summary of predicted microbial communities involved in the degradation of specific neutral and acidic polysaccharides. **(C)** Integrated predictions and observations of microbial degraders of neutral and acidic polysaccharides.

To test whether different microbial groups from pustular mats can degrade structurally and chemically diverse polysaccharides likely present in these mats, we enriched Shark Bay pustules on several commercially available polysaccharides selected to mimic key elements of mat EPS. We first crushed the original pustules into a slurry and incubated the slurry for 2 weeks in seawater medium. The medium was amended with one of seven commercially available polysaccharides (Figure 6) and cultures were either incubated in the dark to prevent the growth of cyanobacteria or incubated without any polysaccharide amendments in the light to ensure the continued production of native EPS by cyanobacteria. Cellulose was the analog for linear polymers of glucose with β-1,4 linkages because glucose was among the most abundant monosaccharide in the hydrolysates of the cyanobacterial EPS (Figure 4A). Laminarin, a β-1,3-glucan with 1,6 branches typically found in brown algae, was chosen as another glucan. The detection of uronic acids and sulfate functional groups confirmed the production of acidic polysaccharides by Cyanobacteria (Figures 3, 4, 7A). Chondroitin sulfate, a glycosaminoglycan composed of repeating glucuronic acid and N-acetylgalactosamine residues that is variably sulfated at the C-4 or C-6 positions, was used as the analog for acidic polysaccharides that contain N-acetyl sugars, uronic acids and are sulfated. Pectin—a heteropolysaccharide with a homogalacturonan backbone of galacturonic acid that can be methyl-esterified or acetylated but generally lacks sulfate groups—was used as the analog for acidic polysaccharides containing uronic acids without sulfate moieties. Agar—consisting of largely unbranched agarose (a repeating disaccharide of galactose and 3,6-anhydrogalactose) together with branched agaropectin that is variably sulfated and methylated—was chosen because it is a marine polysaccharide containing mostly neutral sugars and a relatively low proportion of acidic groups. Xylan, a hemicellulose with a β-(1,4)-linked xylose backbone that is commonly acetylated and branched with arabinose or glucuronic acid side chains, was included as the analog for acetylated polysaccharides. Finally, chitin, a linear β-(1,4)-linked homopolymer of N-acetylglucosamine that is variably acetylated but unbranched and not sulfated, were used as analogs of aminoacetylated polysaccharides.

We compared 16S rRNA gene amplicons from all cultures to those from the raw, untreated pustule, which had been stored in the dark at 4 °C. Cultures that became visibly turbid after 2 weeks yielded an average of 377,306 reads, whereas cultures that showed no visible growth or detectable DNA upon extraction averaged only 49,117 reads. In total, we identified 3,602 ASVs, with the pustule community exhibiting the highest diversity (3,180 ASVs). In contrast, the enrichments on pectin and chitin did not become turbid, and their extracts did not contain detectable DNA and exhibited the lowest diversity, containing just 47 and 58 ASVs, respectively. Because chitin and pectin did not support detectable growth, these amendments were classified as failed enrichments.

**Figure 8:**
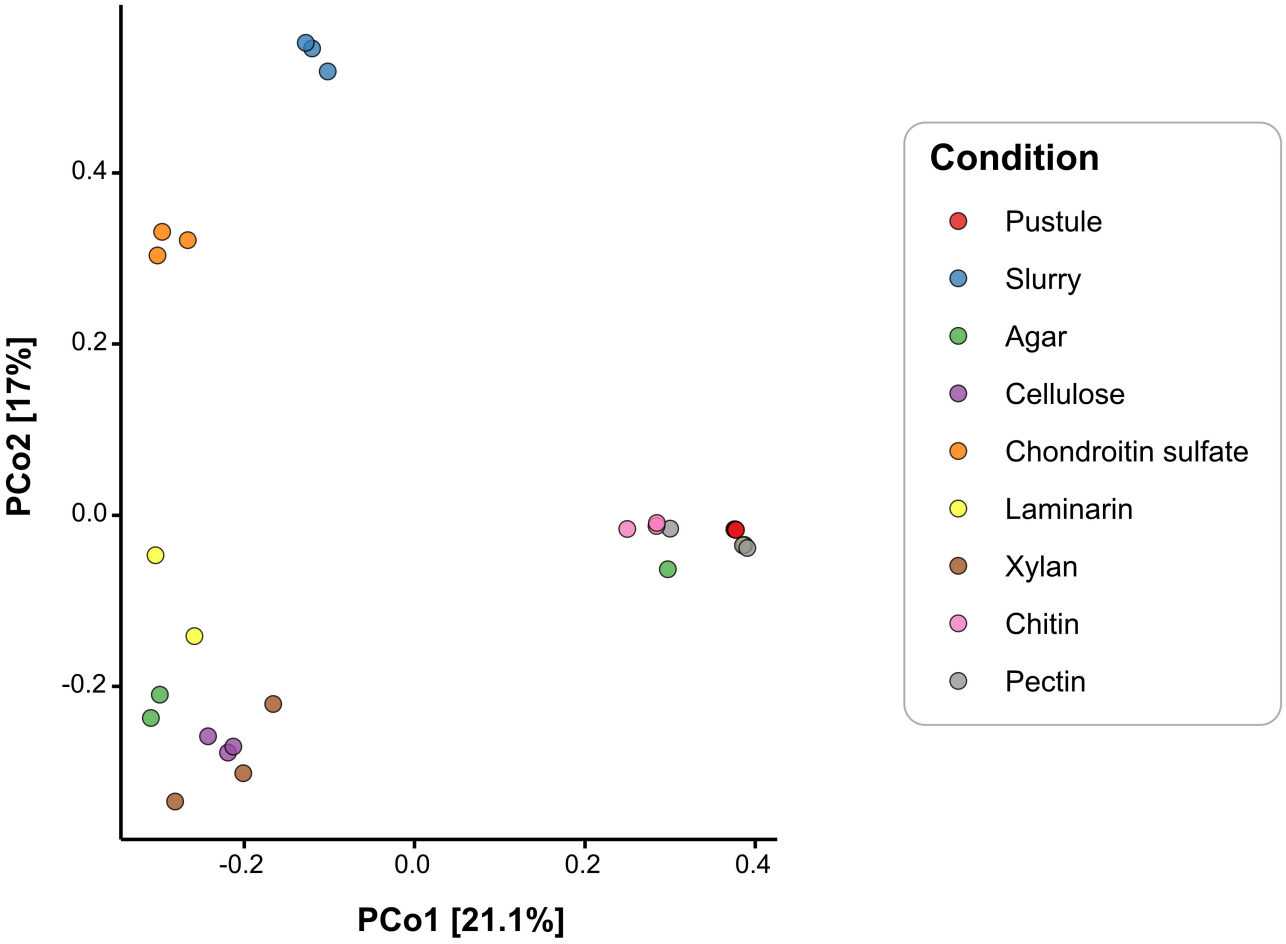
Principal Coordinates Analysis of microbial communities based on Bray-Curtis distances of 3,602 ASVs from pustules, slurry, and enrichment cultures. Colors represent experimental enrichment conditions.

Cyanobacterial ASVs were abundant in the raw pustule and the light-grown slurry but were absent from the dark polysaccharide enrichments that exhibited growth and yielded detectable DNA (Figure 6B). Cellulose, agar, chondroitin sulfate, laminarin, and xylan enriched dark-grown communities that contained primarily Alphaproteobacteria, Gammaproteobacteria, and Bacteroidetes (Figure 6B). Sulfated polysaccharides containing uronic acids, such as agar and chondroitin sulfate, additionally enriched for Planctomycetes (Figure 6B). PCoA ordination grouped cultures enriched on chondroitin sulfate closely with the replicates of light-grown pustule slurry (Figure 8), suggesting that this polysaccharide may share greater compositional and structural similarities with the native EPS than the other tested polysaccharides. Community composition differed among enrichment conditions (*p* < 0.001), suggesting an influence of the substrate type on enrichment outcomes. The communities present in the cultures amended by chitin and pectin did not differ significantly from the pustule (*q* > 0.1), supporting the inference that their community profiles largely reflected residual inoculum DNA rather than enrichment. The lack of growth on chitin and pectin also suggested that these polysaccharides were poor analogs of those present in the native EPS. Conversely, glucan- and xylan-enriched communities (cellulose, laminarin, and xylan) were significantly distinct from the chondroitin sulfate, slurry, and pustule-like group (*q* < 0.01).

Alphaproteobacteria are present throughout pustular mats in Shark Bay, both within and outside the photic zone (Wong et al., 2015). Their MAGs also exhibit the highest alpha diversity in the pustular mat community (Skoog et al., 2022) and, with the exception of MAG 22, primarily contain genes associated with the breakdown of simpler alpha- and beta-glucans (Figure 5; Supplementary Figure 3). These observations and predictions are consistent with the abundance of Alphaproteobacterial ASVs in the enrichments on glucans (i.e., laminarin and cellulose) and xylan (Figure 6B; Supplementary Figure 3). Parvularcula was specifically enriched and is notable given that the anomalously CAZyme-rich Alphaproteobacterial MAG 22 recovered from the Shark Bay pustule belonged to Parvularculaceae family (Supplementary Figure 3). The communities in the pustule and light-grown slurry and the communities enriched on heavily decorated polysaccharides contained fewer ASVs belonging to Alphaproteobacteria, suggesting that most Alphaproteobacteria in pustular mats may be adapted toward utilizing simpler polysaccharides such as glucans and xylan.

Nearly one quarter of the MAGs that represent the community in the native pustular mat belong to Bacteroidetes (Skoog et al., 2022). A high percentage of these MAGs encode the potential to degrade glycosaminoglycans and algal polysaccharides (i.e., sulfate and uronic acid-containing acidic polysaccharides; Figure 5). Consistent with this potential, Bacteroidetes ASVs dominated the community in the light-grown slurry and a greater number of distinct Bacteroidetes ASVs were present in this condition than in the enrichments on any of the commercial polysaccharides. The highest percentages of Bacteroidetes (58.8% and 12.5%) were enriched during growth on chondroitin sulfate and agar. Bacteroidetes ASVs in both the light-grown slurry and enrichments were composed primarily of *Marivirga* (Cytophagales) and *Muricauda* (Flavobacteriales), families that were also represented by Bacteroidetes MAGs (Supplementary Figure 3). These specialists likely outcompete other Bacteroidetes in the presence of acidic polysaccharides and may be enriched in regions of the mat where these specific acidic polysaccharides are produced by cyanobacteria (Skoog et al., 2022) or other microbes.

Gammaproteobacteria MAGs recovered from the pustular mats exhibited metabolic potential for degrading sulfated polysaccharides, including glycosaminoglycans and algal polysaccharides, as well as glucans such as cellulose, suggesting metabolic versatility (Figure 5; Supplementary Figure 3). Enrichment experiments confirmed the presence and growth of Gammaproteobacteria communities in all enrichment cultures, as expected from their predicted degradation potential (Figure 6B). Planctomycetes MAGs present in pustular mats were also predicted to degrade various polysaccharides (Figure 5). Planctomycetes ASVs were detected in the native pustule as well as in the light-grown slurry and the enrichment cultures grown on agar and chondroitin sulfate in the dark. These observations support a role of Planctomycetes in the degradation of acidic polysaccharides (Figures 7B, C). On the other hand, Chloroflexi and some Verrucomicrobia were also predicted to degrade a variety of neutral and acidic polysaccharides (Figures 7B, C) but were not detected in the slurry cultures (Figure 6B). The presence of these taxa in the undisturbed pustule and their absence from the light-grown slurry and dark-amended slurry enrichment cultures suggest that these taxa may be better adapted to the microaerophilic or anaerobic conditions present within intact microbial mats, where oxygen gradients create special ecological niches.

## 4 Discussion

### 4.1 Diversity of EPS in pustular microbial mats

EPS are the most abundant components of microbial mats, where they play key roles in microbial attachment to the sediment-water interface, protect against environmental stresses, and mediate chemical and physical processes in biogeochemical cycling. However, characterization of polysaccharides in cyanobacterial cultures and mats has been challenging due to the changes in growth phases, environmental conditions, and types of cyanobacterial taxa (De Vuyst and Degeest, 1999; Ricciardi et al., 2002; Fischer et al., 2003; Bahat-Samet et al., 2004; Vaningelgem et al., 2004; Panhota et al., 2007). The chemical structure of EPS in marine cyanobacterial mats is mostly unknown, but the polymers made by cyanobacteria are usually very large (>1 MDa), complex and commonly incorporate 6–13 different sugars (including uronic acids, amino sugars, and deoxysugars), in contrast to other bacterial EPS which typically contain less than 4 (Pereira et al., 2009). Compositional and metagenomic analyses show that the extracellular polysaccharides synthesized by cyanobacteria and photosynthetic communities enriched from pustular mats in Shark Bay, Australia, are composed of at least ten monosaccharides, are structurally diverse and are decorated by a variety of functional groups, including acetyl and acidic moieties such as carboxylic groups and sulfate. Additionally, weaker signals of hydroxyl groups and carboxyl bands in cultures exposed to UV compared to those not exposed to UV may be consistent with a greater degree of cross-linking and lower hydration of the EPS (e.g., Antunes et al., 2010; Nikjoo et al., 2021), although this inference remains speculative. Therefore, the composition of monosaccharides, decorations and potentially the extent of cross-linking in EPS depend on the growth conditions and stresses such as UV radiation.

### 4.2 Microbial ecology of EPS degradation may influence community structure

Cyanobacterial EPS can account for up to 24% of cyanobacterial net primary productivity (Bertilsson et al., 2005) and are a major source of extracellular carbon (Aluwihare et al., 1997; Pannard et al., 2016; Stuart et al., 2016) that fuels carbon cycling in many microbial communities (Fogg, 1983). Complete degradation of the compositionally complex EPS in pustular mats requires a broad array of functionally diverse CAZymes: the pustular mats from Shark Bay contain organisms with expansive suites of CAZymes and microbial groups that can degrade various polysaccharides. Studies of EPS distribution in stratified, laminated microbial mats reveal that their abundance varies with depth (Che et al., 2001; Rougeaux et al., 2001; Decho et al., 2005; Braissant et al., 2009). In contrast, pustular mats are irregularly laminated and poorly stratified (Figure 1C), so the abundance and composition of EPS may not only vary with the depth from the photic zone, but also with the distance from cyanobacteria, the primary producers of EPS in these systems. Skoog et al. (2022) hypothesized the association between Cyanobacteria—primary producers of sulfated EPS—and Verrucomicrobia and Bacteroidetes, which possess the potential to degrade these polysaccharides. Here, we provide evidence that the same microbial groups (i.e., Bacteroidetes, Chloroflexi, Verrucomicrobia, Planctomycetes, and Hydrogenedentes; Figure 7) can also degrade acidic polysaccharides that contain uronic acids. Fewer Alphaproteobacteria are capable of degrading acidic polysaccharides, but members of this class can degrade simpler polysaccharides both in proximity to and further from cyanobacteria (Figure 5; Wong et al., 2015). In the same vein, organisms located deeper within the mat or further from Cyanobacteria, such as Gammaproteobacteria (Wong et al., 2015), appear to lack most CAZymes involved in the degradation of various decorated and more complex polysaccharides. In this manner, the complexity of polysaccharides in EPS may shape the structure and function of microbial communities in mats.

CAZymes detected in Bacteroidetes, Verrucomicrobia, Planctomycetes, and Chloroflexi (Anaerolineae) and the distinct enrichment of Bacteroidetes and Planctomycetes on agar and chondroitin sulfate support specialization of these organisms in the degradation of acidic polysaccharides. These findings are consistent with the known role of Bacteroidetes as degraders of complex polysaccharides in other systems such as the human gut (Thomas et al., 2011; Foley et al., 2016; Li et al., 2017; Lapébie et al., 2019; Sun et al., 2021; Grondin et al., 2022) and marine systems (Bengtsson and Øvreås, 2010; Martinez-Garcia et al., 2012; Erbilgin et al., 2014; Bayer et al., 2018; Sichert et al., 2020; Robbins et al., 2021; Ye et al., 2022). Verrucomicrobia are found in carbohydrate-rich systems such as human and animal guts (Lin et al., 2016; Fujio-Vejar et al., 2017), rice paddies (Chin et al., 2001), organic-rich lakes (He et al., 2017) and marine macro- and microalgae (Yoon et al., 2008; Bashenkhaeva et al., 2015; Vollmers et al., 2017), and are functionally and numerically important members of bacterioplankton communities that degrade recalcitrant sulfated algal polysaccharides including fucoidan (Sichert et al., 2020; Orsi et al., 2016). The detection of fucose—the primary monosaccharide in fucoidan—in the EPS from all cultures and growth conditions suggests that fucose-containing polysaccharides are an important component of mat EPS under a range of environmental conditions. If, like fucoidan, these polysaccharides are acidic, they may feed Bacteroidetes, Verrucomicrobia, Planctomycetes, and Anaerolinae in pustular mats.

Hydrogenedentes, Chloroflexi, and Verrucomicrobia were notably absent from enrichment cultures, but were present in the intact pustule. The growth of these groups in natural pustular mats is likely influenced by additional factors such as oxygen availability. The enrichment cultures were aerated, which likely suppressed the growth of obligate anaerobes such as Anaerolineae that can degrade sugars in salt marsh ecosystems (Payne et al., 2025) and are core members of cellulolytic bioreactor communities (Podosokorskaya et al., 2013; Xia et al., 2016). Microbial members of the Hydrogenedentes candidate phylum (*Ca*. Hydrogenedentota) are typically present at very low abundances in natural mats (< 1%), making them challenging to enrich in culture (Skoog and Bosak, 2024). Alphaproteobacteria, Gammaproteobacteria, Myxococcota, Chlamydiae, and the Candidate phylum Sumerlaeota (formerly BRC1, Skoog and Bosak, 2024) in pustular mats possess a lower diversity of CAZymes and appear to be capable of degrading primarily simpler polysaccharides, such as alpha-glucans and beta-glucans (Figures 5, 7).

### 4.3 Microbial stress adaptations likely shape EPS dynamics and microbial activity

Cyanobacterial EPS plays an important role in protecting microbial mats from environmental stresses such as desiccation, viral infection, and UV exposure (Grilli Caiola et al., 1993; Caiola et al., 1996; Baba et al., 1988; Tamaru et al., 2005; Ghosh et al., 2009; Ozturk and Aslim, 2010; Rossi and De Philippis, 2016; Skoog et al., 2022; Laroche, 2022). Here, we detect xylose exclusively in the EPS produced by cyanobacteria exposed to UV wavelengths. Xylose is the substrate for the synthesis of the polysaccharide xylan, an intermediate in the synthesis of hemicellulose (Mohagheghi et al., 1988). If some of the xylose is present in xylan-like polysaccharides, the enrichments of Alphaproteobacteria and Gammaproteobacteria in the cultures amended by xylan suggests that these organisms may play a more prominent role in EPS degradation in pustular mats exposed to UV radiation. Xylose, through metabolism via the pentose phosphate pathway, can also contribute to the synthesis of sedoheptulose-7-phosphate, a key precursor for mycosporine-like amino acids (MAAs; Horecker et al., 1956; Balskus and Walsh, 2010). MAAs are UV-absorbing compounds produced by organisms such as lichens, fungi, algae and cyanobacteria upon exposure to UV radiation (Garcia-Pichel et al., 1993; Portwich and Garcia-Pichel, 1999; Balskus and Walsh, 2010; Geraldes and Pinto, 2021). Additionally, the detection of amine groups only in the EPS extracted from the UV-exposed cyanobacterial enrichment cultures (Figure 3), may suggest the presence and potential role of amine functional groups in resistance to UV stress. These identified amine groups may be from MAAs.

To combat osmotic stresses, organisms in hypersaline environments such as Shark Bay can import (“salt-in-cytoplasm” mechanism) or produce compatible solutes such as trehalose, which is also a disaccharide sugar (Brown, 1976; Galinski and Trüper, 1994; Kunte, 2006; Goh et al., 2011; Youssef et al., 2014). Trehalose was previously identified as an osmolyte in cyanobacteria isolated from Shark Bay stromatolites (Goh et al., 2010). Each Cyanobacterial MAG analyzed here possesses the genomic capacity to synthesize trehalose (Supplementary Figure 1). Additionally, several organisms represented by other MAGs also possess trehalose transporters for the uptake of this osmoprotectant (Skoog et al., 2023). The identification of trehalose-degrading enzymes (i.e., GH13) in Cyanobacteria, Verrucomicrobia, Bacteroidetes, Alphaproteobacteria and Planctomycetes suggests that various community members leverage trehalose, created potentially as a stress response mechanism, to generate energy for their own metabolic needs by breaking down trehalose into glucose monomers for catabolism (Boos et al., 1990; Sakaguchi, 2020; de Graaff et al., 2021). This further emphasizes the interwoven nature of EPS cycling, community structure and stress response.

### 4.4 Influence of acidic polysaccharides on calcification

Variably charged polysaccharides and proteoglycans underpin the calcification in microbial mats (Disnar and Trichet, 1981; Défarge et al., 1994; Kawaguchi and Decho, 2002; Skoog et al., 2022) and eukaryotes such as mollusks, coccolithophores and foraminifera (Giuffre et al., 2013; Arias et al., 2001; Arias and Fern?ndez, 2008). In coccolith-associated polysaccharides, uronic acids have been implicated in initiating calcite nucleation and precipitation (De Jong et al., 1976; Fichtinger-Schepman et al., 1981; Borman et al., 1982, 1987; Lee et al., 2016; Gal et al., 2016), whereas sulfated polysaccharides (e.g., sulfated galacturonomannan) seem to play a larger role in downstream calcification processes (Borman et al., 1982; Marsh et al., 2002). Chemically distinct zones of the aragonitic shell of the cephalopod *Nautilus pompilius* also contain varying amounts of carboxylate and sulfate moieties (Bonucci, 2009; Petrochenkov et al., 2018).

Various observations correlate the calcification in marine mats to the degradation of acidic EPS. Biochemical analyses of microbial mats and laboratory-cultured cyanobacterial EPS (*Schizothrix* sp.) associated high concentrations of uronic acids and carbohydrates with unlithified EPS layers (Kawaguchi and Decho, 2002). Calcification in the stromatolitic mats in Highborne Cay, The Bahamas, occurs in the zones of sulfate reduction, suggesting that the degradation of EPS under anoxic conditions facilitates the nucleation and precipitation of carbonate (Visscher et al., 2000). Similarly, calcification in kopara occurs on the degraded cyanobacterial EPS in the mats formed by the benthic cyanobacterium *Phormidium* (Phormidesmiaceae; Défarge et al., 1994) and the calcified zones in pustular mats from Shark Bay contain fewer active cyanobacteria and sulfated polysaccharides, suggesting the inhibition of calcification by sulfated polysaccharides produced by cyanobacteria (Skoog et al., 2022). This correlation may be understood in the context of mineral saturation: highly charged acidic polysaccharides such as heparin stimulate the nucleation of calcite only in highly supersaturated solutions, whereas more neutral polysaccharides are more conducive to nucleation at lower degrees of saturation (Giuffre et al., 2013). Thus, in marine microbial mats calcium carbonate precipitation and microbial mat lithification may critically depend on the activity of Bacteroidetes, Verrucomicrobia, Planctomycetes, and Chloroflexi—microbes that are capable of degrading acidic polysaccharides excreted by cyanobacteria. These spatial and compositional correlations support the hypothesized role of EPS degradation in facilitating carbonate precipitation. Direct measurements of nucleation dynamics, saturation states as functions of different substrates and microbial community composition and activity are needed to validate this proposed mechanism and represent an important next step for future experimental work.

### 4.5 Methodological considerations and future perspectives

It is important to acknowledge certain limitations of this study. First, inferring precise substrate preferences from CAZy families is challenging due to their broad functional diversity. Thus, the annotations presented here should be regarded as putative rather than definitive functional assignments. Furthermore, a promising direction for future work would be the identification and characterization of polysaccharide utilization loci, particularly in Bacteroidetes, to strengthen the functional link between genomic potential and substrate utilization in pustular mats. Secondly, it is also important to note that the EPS fractions analyzed in this study were obtained using a NaCl/heat extraction and ethanol precipitation method, which is effective for recovering soluble (S-EPS) and loosely bound (LB-EPS) polymers while minimizing contamination from cell lysis, but largely excludes the tightly bound (TB-EPS) fraction. The TB-EPS fraction, which remains closely associated with the cell surface, is often enriched in acidic groups such as uronic acids and likely plays a critical role as the immediate interface for processes such as mineral nucleation. However, future work examining exactly how and to what extent acidic polysaccharides in this EPS fraction influence microbe–mineral interactions is needed in order to better understand their potential role in this process. Advancing our understanding will require characterizing EPS composition and mineral nuclei at the scale of individual cells to more clearly resolve these processes. Furthermore, the monosaccharide profiles and UV-induced shifts reported here represent only a subset of the total EPS pool, and our conclusions may underestimate the ecological importance of bacteria specialized in degrading polymers enriched in TB-EPS. Future studies employing complementary extraction approaches will be necessary to capture TB-EPS and provide a more comprehensive understanding of EPS composition, ecological function, and contributions to carbonate mineralization.

## Data Availability

The datasets analyzed for this study can be found in the Joint Genome Institute IMG/M system under GOLD AP ID Ga0316160. Raw metagenomic sequence data and MAGs are also publicly available on Zenodo (https://doi.org/10.5281/zenodo.3874996). Amplicon sequence reads generated during this study are available from the NCBI SRA under BioProject accession PRJNA826985, with individual run accessions SRX14890888-SRX14890907.

## Supplementary material

Supplementary Figure 1 Presence/absence of genes involved in the production of EPS within three cyanobacterial MAGs. Identified genes include specific glycosyltransferases (GT), general EPS synthesis genes, and genes with associations to specific monosaccharides including mannose (Man), rhamnose (Rham), arabinose (Ara), Cellulose (Cel), galactose (Gal), and fucose (Fuc). Acetyltransferase (AcetylT), sulfotransferase (SulfT), and methyltransferase (MetT) genes involved in EPS modification are also identified.

Supplementary Figure 2 HPAEC-PAD chromatograms of hydrolyzed standards.

Supplementary Figure 3 Distribution of potential polysaccharide substrates of GH and PL identified in each Shark Bay MAG. Colors reflect the number of genes in a given MAG with predicted activities on respective substrates. Genes can be counted more than once if they have more than one potential substrate. Genes from polyspecific families were counted only in the column labeled “polyspecific” (see Methods). The abundance of GH with predicted activity only on glycosides (i.e., not specifically on polysaccharides or oligosaccharides) are shown in the column labeled “glycosides only.” GH and PL with unknown activities are shown in the column labeled “unknown.” Algal polysaccharides include algin, carrageenan, ulvan, fucoidan, and beta-(1,4)-galacturonan. Glycosaminoglycans (GAGs) include chondroitin sulfate, dermatan sulfate, heparin/heparan sulfate, and unidentified GAGs. “Other EPSs” include the known bacterial exopolymers xanthan, gellan, and polygalactosamine.
