# Supplementary figures and images for: Linking microbial ecology to the cycling of neutral and acidic polysaccharides in pustular mats from Shark Bay, Western Australia
Source: Front Microbiol. 2025 Oct 13;16:1684648. doi: 10.3389/fmicb.2025.1684648 (PMC12555021; doi:10.3389/fmicb.2025.1684648)

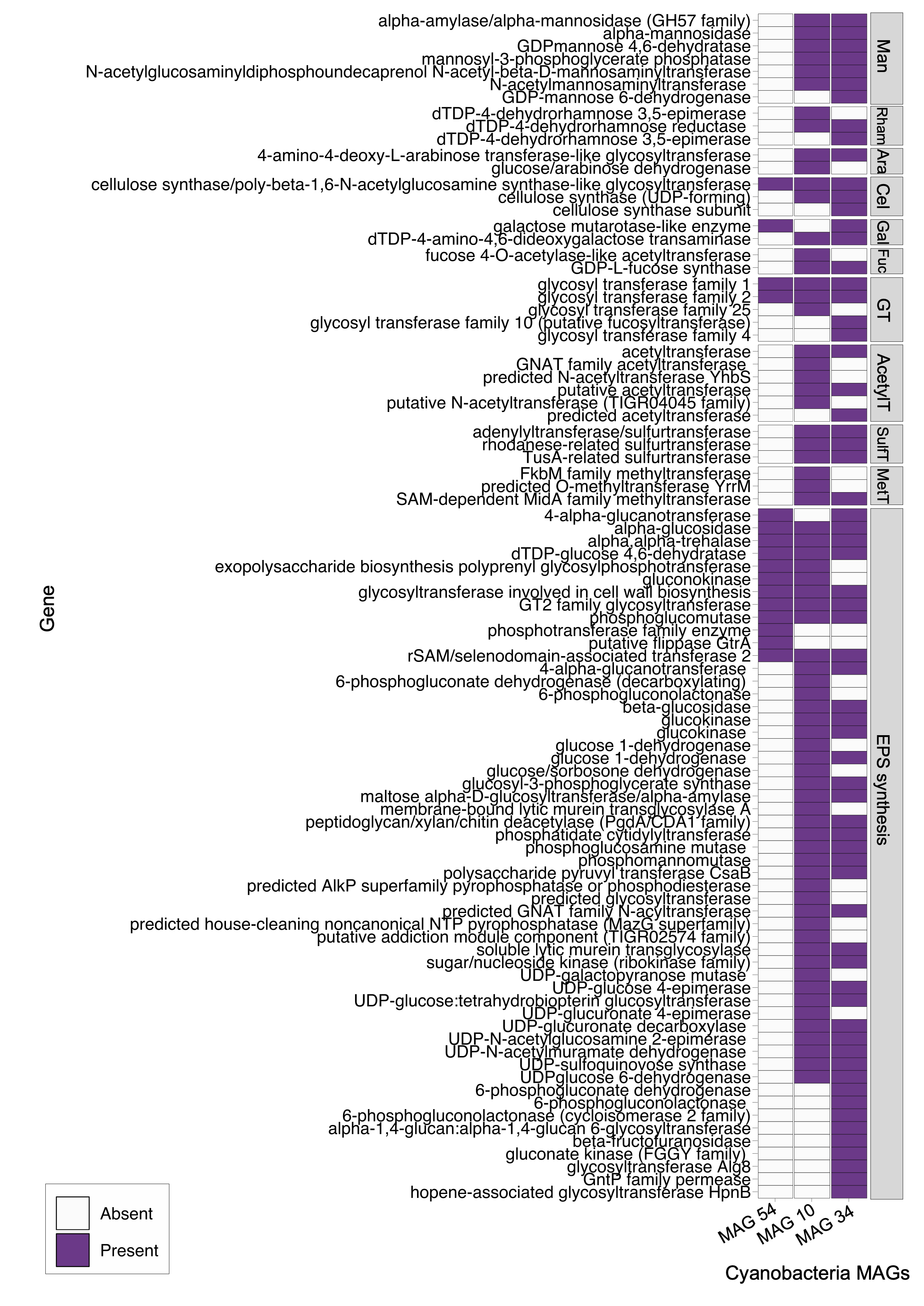

Supplement: Supplementary Figure 1 — Presence/absence of genes involved in the production of EPS within three cyanobacterial MAGs. Identified genes include specific glycosyltransferases (GT), general EPS synthesis genes, and genes with associations to specific monosaccharides including mannose (Man), rhamnose (Rham), arabinose (Ara), Cellulose (Cel), galactose (Gal), and fucose (Fuc). Acetyltransferase (AcetylT), sulfotransferase (SulfT), and methyltransferase (MetT) genes involved in EPS modification are also identified. [file Image_1.png]

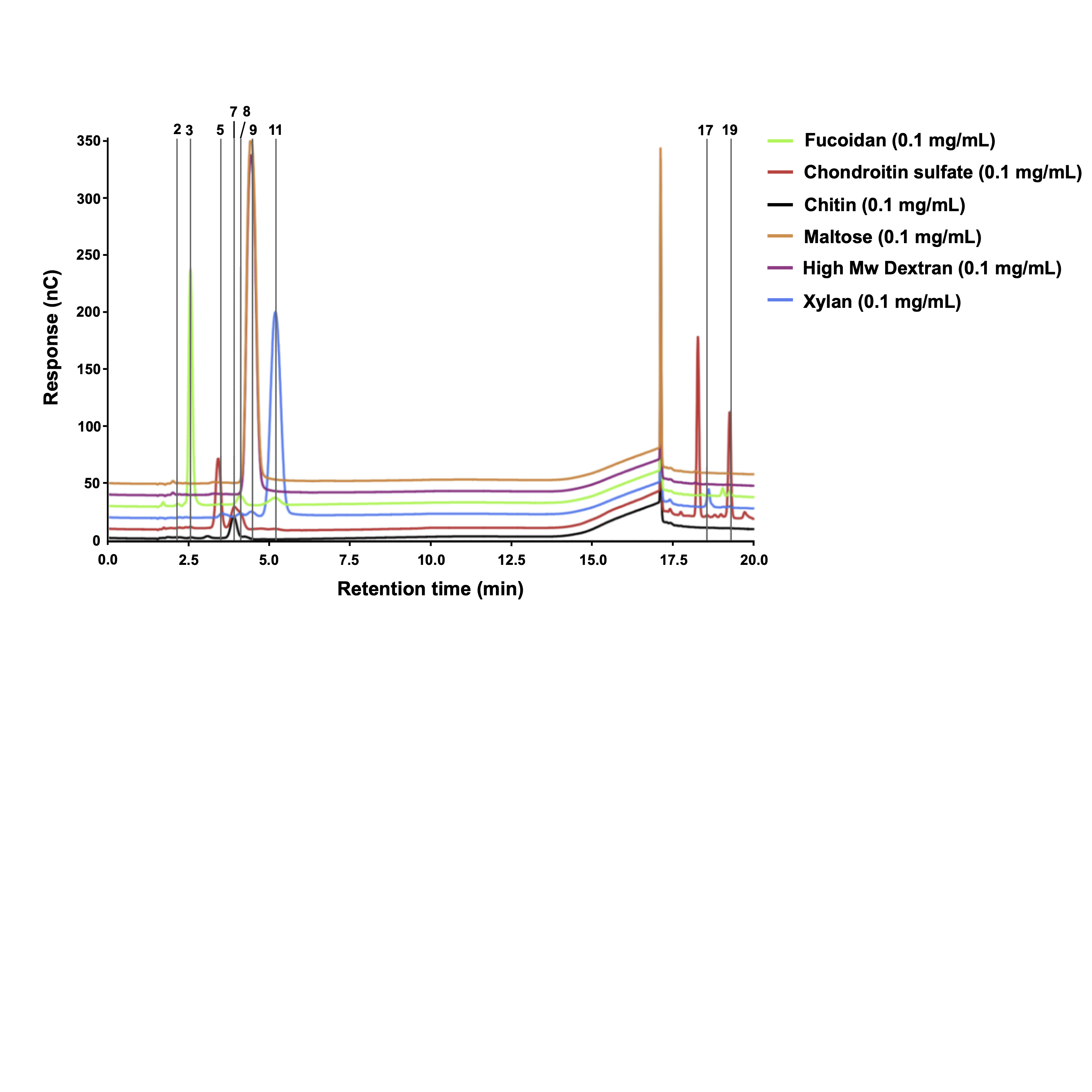

Supplement: Supplementary Figure 2 — HPAEC-PAD chromatograms of hydrolyzed standards. [file Image_2.png]

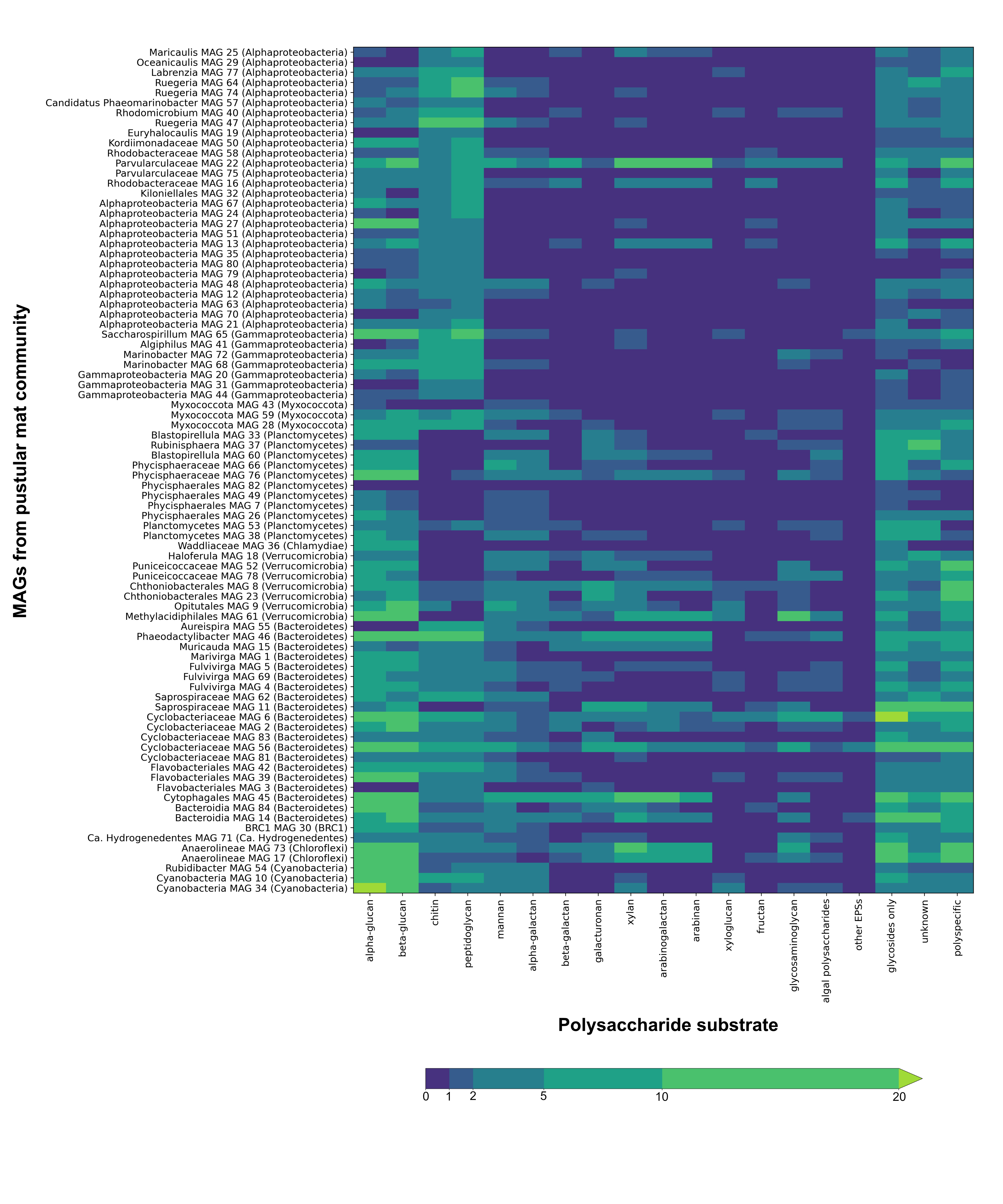

Supplement: Supplementary Figure 3 — Distribution of potential polysaccharide substrates of GH and PL identified in each Shark Bay MAG. Colors reflect the number of genes in a given MAG with predicted activities on respective substrates. Genes can be counted more than once if they have more than one potential substrate. Genes from polyspecific families were counted only in the column labeled “polyspecific” (see Methods). The abundance of GH with predicted activity only on glycosides (i.e., not specifically on polysaccharides or oligosaccharides) are shown in the column labeled “glycosides only.” GH and PL with unknown activities are shown in the column labeled “unknown.” Algal polysaccharides include algin, carrageenan, ulvan, fucoidan, and beta-(1,4)-galacturonan. Glycosaminoglycans (GAGs) include chondroitin sulfate, dermatan sulfate, heparin/heparan sulfate, and unidentified GAGs. “Other EPSs” include the known bacterial exopolymers xanthan, gellan, and polygalactosamine. [file Image_3.png]
